# Direct integration of intensity-level data from Affymetrix and Illumina microarrays improves statistical power for robust reanalysis

**DOI:** 10.1186/1755-8794-5-35

**Published:** 2012-08-21

**Authors:** Arran K Turnbull, Robert R Kitchen, Alexey A Larionov, Lorna Renshaw, J Michael Dixon, Andrew H Sims

**Affiliations:** 1 Breakthrough Research Unit, University of Edinburgh, Crewe Road South, Edinburgh, EH4 2XR, UK; 2 Current address: Yale University School of Medicine, Department of Molecular Biophysics & Biochemistry and Department of Psychiatry, 266 Whitney Ave, New Haven, CT, 06511, USA

## Abstract

**Background:**

Affymetrix GeneChips and Illumina BeadArrays are the most widely used commercial single channel gene expression microarrays. Public data repositories are an extremely valuable resource, providing array-derived gene expression measurements from many thousands of experiments. Unfortunately many of these studies are underpowered and it is desirable to improve power by combining data from more than one study; we sought to determine whether platform-specific bias precludes direct integration of probe intensity signals for combined reanalysis.

**Results:**

Using Affymetrix and Illumina data from the microarray quality control project, from our own clinical samples, and from additional publicly available datasets we evaluated several approaches to directly integrate intensity level expression data from the two platforms. After mapping probe sequences to Ensembl genes we demonstrate that, ComBat and cross platform normalisation (XPN), significantly outperform mean-centering and distance-weighted discrimination (DWD) in terms of minimising inter-platform variance. In particular we observed that DWD, a popular method used in a number of previous studies, removed systematic bias at the expense of genuine biological variability, potentially reducing legitimate biological differences from integrated datasets.

**Conclusion:**

Normalised and batch-corrected intensity-level data from Affymetrix and Illumina microarrays can be directly combined to generate biologically meaningful results with improved statistical power for robust, integrated reanalysis.

## Background

In the clinical sciences, systematic review is a valuable tool to synthesise high-quality empirical evidence from independent investigations in order to determine a consensus view. Such reviews, or meta-analyses have greater statistical power to identify true effects from study-specific artefacts and, as such, are capable of identifying subtle effects that might be missed or deemed insignificant in smaller datasets. In the context of gene-expression analyses, meta-analysis of results from microarray studies has great potential, but also presents significant challenges due to differences between the platforms and analysis approaches employed in each study [1-5]. Direct integration of probe-level expression data from multiple studies is potentially even more powerful, but is further complicated due to differences in the conditions under which each dataset was generated, such as the amplification or labelling method, the scanner used or even just the date on which the samples were processed. A recent comprehensive review found that the aims of different microarray meta-analysis studies were quite distinct, with the majority combining p-values, effect size or ranked analysis, with only 27% (51 studies) seeking to directly merge the data and most of these were studies used the same platform [1]. We and others have previously demonstrated that non-trivial systematic bias or ‘batch effects’ can occur within both Affymetrix GeneChips and Illumina Beadarrays [3,4,6,7], but that they can largely be removed from each with appropriate correction methods.

Gene expression profiling has been applied to many areas of translational cancer research, including identification of new drug-targets, monitoring response to treatment, revealing mechanisms of resistance, and predicting prognosis [8]. Although the majority of datasets are now made publicly available, many studies are limited in size and therefore cannot accurately reflect the general population, as they lack statistical power [9,10]. A consequence of this is that gene signatures generated from a small cohort of patients (the ‘training set’), will never perform as well in subsequent cohorts (‘test sets’) which inevitably have subtle differences in composition of patient or tumour variables. We previously showed that combining several similar Affymetrix datasets leads to a greater overlap in differentially expressed genes and more accurate prognostic predictions [5]. Collection of clinical material often remains the rate-limiting step, particularly with valuable ‘window-of-opportunity’ studies that utilise matched *before*- and *after*-intervention samples from the same patient [6,11-14]. Due to the reduced patient-patient variation, these studies can be highly effective for identifying consistent gene-expression changes, such as the effects of (neoadjuvant) cancer treatment.

The extensive patient- and tissue-diversity inherent in molecular studies of cancer, which often contribute to underpowered studies [9] and confounding [15], mean that it is currently not necessarily critical (or appropriate) to measure gene-expression at the greatest resolution or specificity now offered by exon-arrays and RNA-sequencing. Rather, it may be of greater utility to maximise the number of existing biologically independent observations by combining the growing numbers of datasets in the public repositories, instead of simply generating another small independent dataset with limited statistical power [8].

Previous comparisons of expression measurements derived from Affymetrix and Illumina platforms have reported, ‘generally consistent’ [16], ‘very high agreement’ [17] or ‘correspondence across platforms was high’ [18]. However these studies are often based on titrated or technical replicates rather than clinical samples and have not sought to integrate the intensity-level data directly. Cross-platform analysis of microarray data has previously been shown to be possible and worthwhile, although this has normally been performed using transformed relative values [19], analogous to those from two-colour microarrays and have been shown to result in fold change compression [18].

Considering the fundamental differences in the design of the two platforms, it is not clear whether data derived from Affymetrix and Illumina microarrays can be reliably compared directly. In this study we demonstrate that it is possible to directly combine appropriate datasets at the intensity level to improve statistical power. We show that the inter-platform bias can be sufficiently reduced to expose previously obscured biological variation and that such data correction does not amplify meaningless noise in the results. Despite intrinsic differences between these technologies, suitably similar studies can be directly integrated for robust and powerful meta-analysis.

## Results

### Direct cross-platform integration of MAQC data

**Figure 1:**
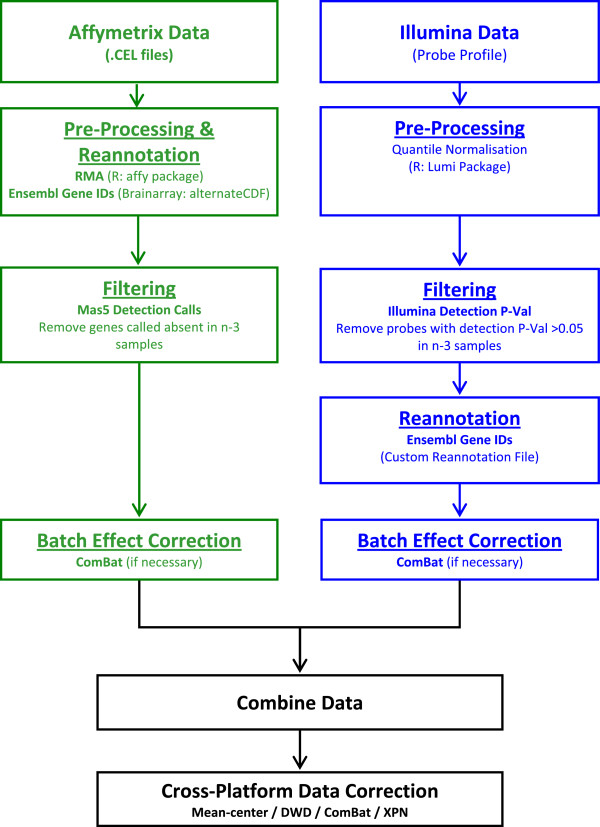
**Summary of the data analysis workflow to assess direct integration of Illumina and Affymetrix gene expression data.** The same/similar processing steps were used wherever possible, Affymetrix in green, Illumina in blue.

**Figure 2:**
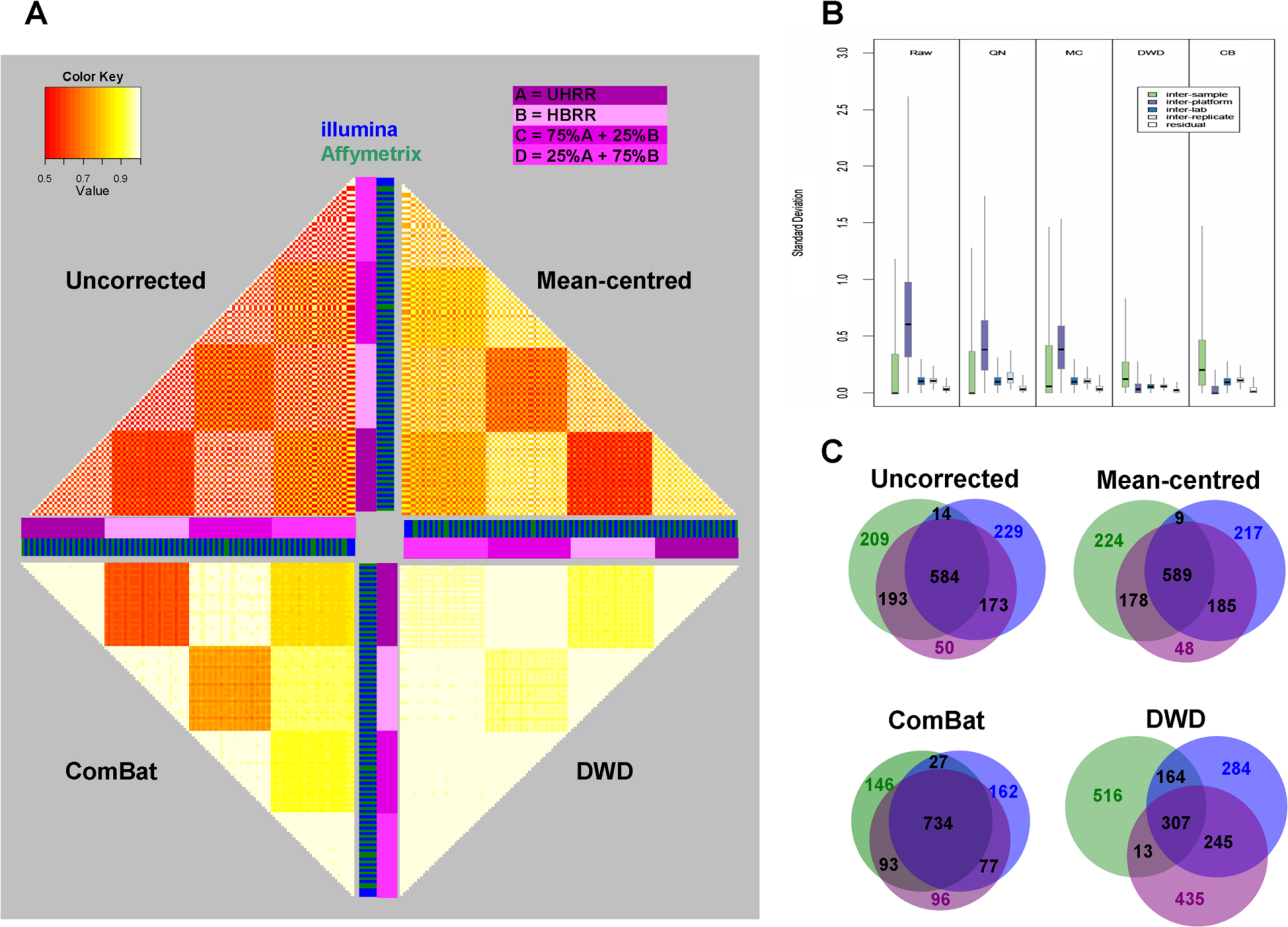
**Affymetrix and Illumina data from the Microarray Quality Control project can be directly integrated.A**) Pairwise Pearson correlation heatmaps (left) demonstrate cross platform bias and the effects of three correction methods, mean-centering, distance-weighted discrimination (DWD) and an Empirical Bayes method (ComBat). R values range from low correlation (red) to high correlation (white) through shades of orange and yellow reflecting the overall similarity of expression profiles based upon biological and platform-specific variation. The shades of purple to pink indicate the samples (A = 100% UHRR, B = 100% HBRR, C = 75% UHRR + 25% HBRR, D = 25% UHRR + 75% HBRR). Samples are ordered by replicate and lab name rather than by platform. Green bars for Affymetrix samples and blue for Illumina samples. Boxplots of correlation coefficients within and between labs are shown in (Additional file 1. **B**) Cross-platform correction minimises technical variation whilst maintaining biological variation and differential expression. **C**) Venn diagrams demonstrate the overlap between the 1000 most differentially expressed genes between the MAQC UHRR and HBRR (A and B samples) using significance analysis of Microarrays (SAM) method with either Affymetrix (Green) or Illumina (Blue) alone, or Affymetrix and Illumina together (Purple).

The Microarray Quality Control (MAQC) consortium [18] investigated the reproducibility of microarray-derived gene expression measurements by assessing performance across platforms, chips, and processing sites using a titration of Universal Human Reference RNA (UHRR) and Human Brain Reference RNA (UBRR). We combined the complete MAQC Affymetrix and Illumina datasets by re-annotating probes on each platform in terms of their Ensembl gene targets (see Methods and Figure 1). As expected, sample A (100% UHRR) replicates from the same platform were found to be more highly correlated with sample C (75% UHRR, 25% HBRR) replicates than the other samples. This was also the case for sample B (100% HBRR) and D (25% UHRR, 75% HBRR) replicates, reflecting their relative biological similarity (Additional file 1A). Without adjustment, correlations between the same samples (A, B, C, or D) processed on different platforms were much lower (R = 0.70-0.77) than the same samples processed only on the Illumina Beadarrays (R = 0.98-1.00; Figure 2A and Additional file 1A) or Affymetrix GeneChips (R = 0.99-1.00).

Adjusting for the platform differences using the mean-centring method [5] provided only a marginal improvement compared to uncorrected data, whilst the Distance Weighted Discrimination (DWD) method [20] suppressed not only the platform-specific bias but also legitimate biological variability between samples (Figure 2 and Additional file 1A). The greatest improvement was observed following correction by ComBat, a method that exploits variance moderation during data adjustment [21]. Similar correlations were found both across and within platforms, suggesting that whilst removing the platform bias, ComBat method retains legitimate biological variation between the biologically distinct samples (Figure 2, Additional file 1A). Another promising method, Cross-Platform Normalisation (XPN) [22], could not be evaluated with these data due to the small number of independent biological replicates.

In addition to correlating expression values, we calculated variance estimates for each of the 15,781 Ensembl genes probed by the two platforms at the inter-sample, inter-platform, inter-laboratory, and inter-chip levels using a nested analysis of variance described in methods (Figure 2B). As expected, and in agreement with the correlation analysis, the difference between the platforms was responsible for the majority of the overall variance in raw (58%), quantile-normalised (47%), and mean-centered (44%) expression data. Inter-platform variance was significantly reduced by both DWD and ComBat, to 15% and 7% of the total, respectively. Consistent with the correlation analysis, the DWD method also substantially reduced inter-sample variance, which is likely to obscure differences between the samples (Figure 2B and methods). Conversely, the ComBat method slightly increased inter-sample variance, potentially uncovering meaningful biological differences between the UHRR/UBRR titrations.

To examine the effects of cross-platform integration on the identification of genes differentially expressed between UHRR and HBRR, we analysed Affymetrix and Illumina data both separately and as a combined dataset. Differential expression was assessed using the Significance Analysis of Microarrays (SAM) method [23], identifying the top 1000 differentially expressed genes and comparing the resulting gene-lists, as described previously [5]. Analysis of the 60 combined Affymetrix plus Illumina HBRR and UHRR samples together, resulted in lower false discovery rates and a greater number of statistically significant differentially expressed genes (Additional file 1B) than when the Affymetrix or Illumina (15 ‘A’ and 15 ‘B’) samples were analysed separately. There were also many more overlapping genes in the combined analysis and either of the platforms following cross-platform correction, again with ComBat performing best (Figure 2C). The overlap of differentially expressed genes identified by samples processed on either of the two platforms independently (15 ‘A’ and 15 ‘B’ samples) was also much more consistent following ComBat, than DWD or mean centering correction (Additional file 1C). Taken together, these results indicate that combining data across the two platforms increases specificity and reduces the number of predicted false positives, suggesting improved statistical power.

### Increasing statistical power through integration of clinical datasets

**Figure 3:**
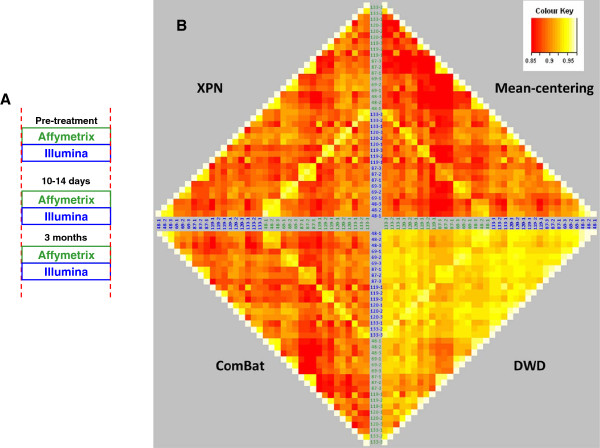
**Clinical samples processed on Affymetrix and Illumina platforms.A**) Experiment Layout of the overlapping 18 matched clinical breast cancer samples from 6 patients from similar Affymetrix and Illumina datasets studying the of the effect of Letrozole in the neoadjuvant setting. **B**) Pairwise Pearson correlation heatmaps (left) from 18 matched clinical breast cancer samples from 6 patients demonstrate cross platform bias and the effects of three correction methods, mean-centering, distance-weighted discrimination (DWD), an Empirical Bayes method (ComBat) and cross platform normalisation (XPN). R values range from low correlation (red) to high correlation (white) through shades of orange and yellow reflecting the overall similarity of expression profiles based upon biological and platform-specific variation. The inner diamond represents the matched samples from the two platforms. Each patient sample is numbered as untreated (−1), 14 days (−2) and 3 months (−3) post treatment. Uncorrected data is NOT shown (to show it on the same colour scale as the other plots would not demonstrate the differences between the correction methods).

In order to evaluate the feasibility of directly comparing intensity level gene expression of clinical samples processed separately on the two platforms, we first generated a new dataset of Illumina Beadarray data from RNA derived from breast tumour samples that were assessed as part of a larger published study using Affymetrix GeneChips [13,24,25] (Figure 3A). These samples comprised matched baseline, two-week, and three-month primary breast tumours from 6 patients with a clinical response to neoadjuvant Letrozole. As with the MAQC data, pairwise Pearson correlations of samples processed on the two platforms were significantly increased following correction with the ComBat method, which again outperformed mean-centering and DWD by maintaining variation between biologically independent samples (Figure 3B and Additional file 2A-C). A fourth method, cross platform normalisation (XPN) [22] generated similar results to ComBat, although Pearson correlations for the majority of matched samples across both platforms were marginally higher (Additional file 2A-C). In addition, a greater number of pairs of Affymetrix and Illumina samples clustered together with the XPN method than with ComBat (Additional file 2E).

**Figure 4:**
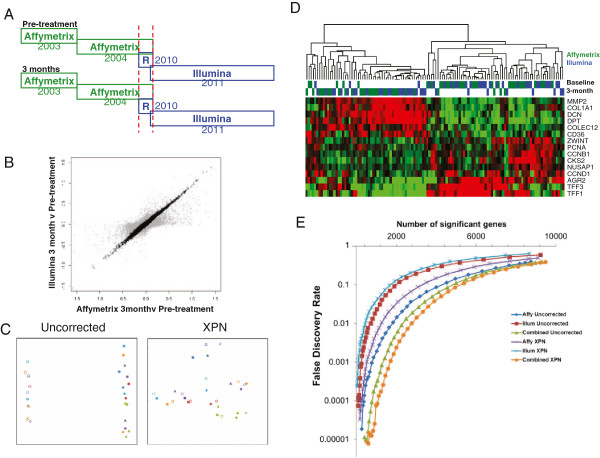
**Integration of partially overlapping Affymetrix and Illumina datasets.A**) Relationship between the baseline and 3 month samples processed on Affymetrix and Illumina platforms. R = repeated samples processed on Illumina BeadChips **B**) Scatterplot demonstrating the fold changes between the Affymetrix and Illumina datasets before (grey) and after XPN correction (black). **C**) Multidimensional scaling plots before and after XPN correction demonstrating the relationship between overlapping samples (circles = baseline, squares = 3 months post treatment with Letrozole, open symbols = Affymetrix, filled symbols = Illumina, triangles = Illumina repeated samples, different colours represent different patients). **D**) Hierarchical clustering and heatmap based on published list of genes identified as most changed between baseline and 3 month samples in patients treated with neoadjuvant Letrozole. Colour bar indicates the platform the sample was processed on with Affymetrix in green and Illumina in blue. **E**) Effect of cross-platform Integration and correction on differential gene expression analysis. Plot shows the relationship between the estimated false discovery rate relative to the number of significant differentially expressed genes identified using SAM analysis of Affymetrix and Illumina datasets independently and when combined both before and after XPN correction. Venn diagrams showing the overlapping genes between the 1000 most differentially expressed genes using the SAM method are available in Additional file 3.

We next expanded the cross-platform dataset with 48 new Illumina baseline and matched three-month samples from 24 independent patients to give a total of 60 Illumina samples to compare with 60 Affymetrix samples from the original dataset. All patients and tumours had similar characteristics and were shown to clinically respond to 3 months of neoadjuvant Letrozole treatment with tumour ultrasound measurements showing a stable volume reduction of 70% over the three-month period. The twelve samples common to both microarrays were retained (Figure 4A). It was necessary to correct for batch effects within the platforms due to date of sample processing using ComBat as described previously [3-5]. Without cross-platform correction, plotting the fold changes between baseline and three-month samples across the two platforms results in reasonable concordance (R = 0.68), however following XPN correction we see a dramatic improvement in the correlation of fold changes (R = 0.99) demonstrating that XPN has greatly reduced the variation between both platforms while maintaining a sufficient range of highly-concordant fold changes to account for biological variability (Figure 4B). Multidimensional scaling (MDS) demonstrated that the samples common to the Affymetrix and Illumina datasets cluster together and that intra- and inter-platform batch effects have been minimised (Figure 4C). Prior to XPN correction samples from the Affymetrix and Illumina datasets form independent clusters, however after correction baseline samples from the same patient cluster closely together as do the three-month samples from the same patient. XPN correction significantly reduces the bias between samples from different platforms, but the baseline and three-month samples from the same patients still cluster independently, indicating that the true biological differences (due to treatment) are maintained. The standard deviation across genes for all baseline or three-month samples was higher in Affymetrix than Illumina, but was dramatically increased after combining the data. Correction with either ComBat or XPN reduced variation to a level similar to that seen in either dataset independently, further suggesting that gene-wise cross-platform bias is reduced, while true biological variation is maintained (Additional File 2D). When all samples of the combined XPN-corrected dataset were clustered by a published list of genes identified as most changed in response to neoadjuvant Letrozole [13,24] the baseline and three-month samples clustered together regardless of platform (Figure 4D).

Increasing sample number by integration of the Affymetrix and Illumina datasets resulted in the identification of a greater number of significantly differentially expressed genes using pairwise SAM (i.e. there was greater consistency of the changes between baseline and three-month samples from the same patients) at a given false discovery rate (Figure 4E). Interestingly, correction of the combined data by XPN showed only minor improvement compared with uncorrected data in a pairwise SAM analysis with an impressive 93.8% overlap of genes (Additional file 3A). However, when a non-pairwise SAM method was used (i.e. two unmatched groups: (i) all baseline samples and (ii) all three-month samples), XPN correction of the integrated data was essential (Additional file 3B&C). There was an impressive 90% overlap of common differentially expressed genes following XPN correction when comparing the baseline samples from one platform with the three-month samples from the other. By contrast, the overlap between baseline and three-month groups in each dataset (Affymetrix or Illumina) independently was only 42.4% (Additional file 3A&B). Finally, comparing the uncorrected Affymetrix baseline versus Illumina three-month samples (and vice versa) with the XPN-corrected equivalent resulted in a very poor overlap (12.1%), indicating the importance of XPN correction for robust differential gene expression of cross-platform integrated datasets.

### Published Affymetrix and Illumina datasets can be successfully integrated

**Figure 5:**
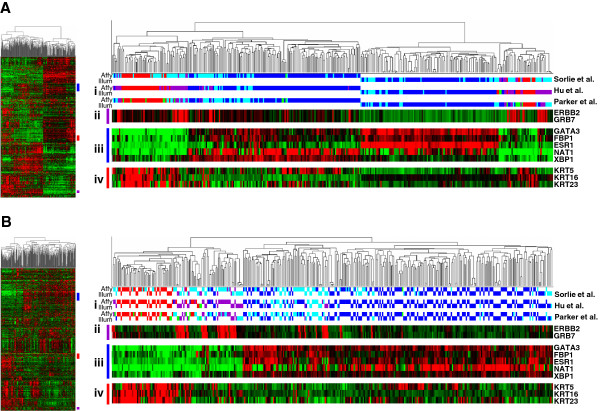
**Comparison of primary breast tumour gene expression profiles generated on Affymetrix and Illumina platforms.** The Nadiri et al. [26] study used Illumina WG6v1 BeadChips, whilst the Desmedt et al. dataset [27] was generated with Affymetrix HG-U133A arrays. **A**) Before cross-platform correction. **B**) After XPN. Hierarchical clustering of tumours is based upon the 500 most variable genes (thumbnails show all genes). i) Subtypes were assigned by three methods Sorlie *et al.* (2003) [5], Parker *et al.*[28] and Hu *et al*. [29]. Red = basal, purple = ERBB2, blue = luminal A, light blue = luminal B, green = normal-like. Clusters of genes associated with the subtypes are highlighted as follows; ii) ERBB2 gene cluster, iii) luminal gene cluster, iv) basal gene cluster.

Two publicly available non-subtype specific primary breast cancer datasets of comparable size and composition (Nadiri *et al.*[26] n = 153 on Illumina WG6v1 and Desmedt *et al.*[27] n = 198 on Affymetrix HGU133A) were assigned to molecular subtypes using centroids from the intrinsic gene signatures of Sorlie *et al.* (2003) [5], Parker *et al.*[28], and Hu *et al*. [29]. This was performed on each dataset independently and then both datasets were combined, both before and after XPN correction. Clustering the integrated data before correction resulted in two distinct clusters representing the two datasets, highlighting the platform-specific systematic bias (Figure 5). Following XPN correction the integrated data clustered based on true biological differences with two clear clusters representing the basal/Her2 intrinsic subtype and the luminal subtype for each of the intrinsic centroids (Figure 5). Assignment of molecular subtype was highly consistent (Sorlie: 96.6%, Hu: 94.9% and Parker: 96.6%) between uncorrected and XPN-corrected datasets, further suggesting that the XPN correction method does not adversely affect the biological interpretation of the data.

Once again, increasing sample number through integrating datasets results in a greater number of significantly differentially expressed genes, between the Sorlie *et al.* basal and luminal-A or the more subtle comparison of luminal A and luminal B subtype samples, at a given FDR (Additional file 4). Uncorrected integrated data performs poorly in comparison to the integrated data after XPN correction or indeed to either dataset independently.

## Discussion

The biggest obstacles to the direct comparison of data obtained from different microarray platforms are differences in the sequence and the number of probes that target each transcript. Many studies simply use the most highly or variably expressed probe to represent a gene, despite evidence that some probes hybridise to multiple genes and others have out-dated or incorrect annotation [30-34]. Limiting integration of data to only those genes where the probe sequences are identical, or comparing measurements simply based upon the official gene symbol would severely restrict our ability to evaluate whether data from different platforms can be directly integrated. For this reason, probes were re-annotated in this study using alternative CDFs [32] for Affymetrix and a validated composite look-up list for Illumina [35].

The microarray quality control (MAQC) project declared that expression values generated on different platforms cannot be directly compared because unique labelling methods and probe sequences will result in variable signals for probes that hybridize to the same target [18]. However in the interests of making the best use of published data on valuable clinical material, we asked whether it would be reasonable to integrate Affymetrix and Illumina data in the interests of improving statistical power and unearthing true biological findings. It has previously been shown that robust classifiers developed using data generated from one platform can accurately predict the phenotype of samples assessed on a different platform [36]. In this study we demonstrate that it is possible to combine Affymetrix and Illumina gene expression data for meaningful integrative reanalysis. As we have previously demonstrated for either platform alone, integration of microarray data should only be performed with appropriately similar datasets [3-5], although exactly where the similarity threshold lies is an important consideration that is still to be determined.

During our analyses we found the Distance Weighted Discrimination (DWD) method [20], which has been used for cross-platform normalisation in a number of published studies (cited by more than 50), inadequate in terms of its ability to remove technical noise and preserve biological variability. Perhaps this method is best suited to transformed data such as that generated by two-colour cDNA studies. We used relatively strict filter-thresholds in our analyses, including conservative detection p-values to limit the analysis to clearly expressed genes as a previous meta-analysis approach found low or intermediate expressing genes to have poorer inter-platform reproducibility than highly expressed genes [14]. Another recently published comparison of cross-platform normalization methods also found XPN to have the highest inter-platform concordance [37]. Like our study this focused on direct adjustment approaches, where the major batch effect (platform used) is clearly identifiable rather than surrogate variable analysis (SVA) approaches [38,39], which look at latent or unknown variables, such as when samples are processed on different days, in different groups or by different people. Direct integration approaches are only appropriate for small numbers of highly similar datasets specifically selected to answer clearly defined questions, as opposed to recent global survey-based approaches used to identify common tissues or expression profiles across all available datasets [40-42]. Whilst integrating data across platforms increases the number of samples, it also has an impact on the number of genes represented. Genes may be ‘lost’ at the reannotation stage if not present on both arrays. Therefore integration is a trade-off between increased sample numbers and decreased gene number. Sample numbers are perhaps the biggest factor in the reliability of microarray studies. Ein-Dor *et al.* suggested that thousands of samples are needed to generate a robust gene list for predicting outcome in cancer [9]. The overlap of differentially expressed genes between single and integrated Affymetrix and Illumina datasets was found to be high, although it should be remembered that it has previously been demonstrated that greater biological reliability is seen between studies at the pathway, rather than individual gene level [8].

## Conclusion

In this study we sought to evaluate whether it is reasonable to directly combine appropriate Affymetrix and Illumina datasets for reanalysis. We found that despite fundamental differences in the technology, data from these platforms can legitimately be combined at the normalised and corrected intensity level, rather than the fold change level for robust reanalysis with improved statistical power than the original datasets alone.

## Materials and Methods

### Data generation

Affymetrix gene expression data was generated from primary breast tumour core biopsies before, 10–14 days after and approximately 3 months following neoadjuvant Letrozole treatment as part of a previously described clinical study [13,25]. The research was carried out in compliance with the Helsinki Declaration, with all patients giving informed consent to be included in the study which had been approved by the local ethics committee (LREC; 2001/8/80 and 2001/8/81). RNA was extracted, amplified and labelled as previously described [25], before hybridisation to HGU-133A GeneChips (Affymetrix) according to the standard protocol. RNA from a subset of 18 samples (baseline, 10–14 days and 3 month samples from 6 patients defined as clinical responders to treatment) used in the aforementioned study [13,25] was then amplified using the WT-Ovation FFPE System Version 2 (NuGEN), purified using the Qiaquick PCR Purification Kit (Qiagen), biotinylated using the IL Encore Biotin Module (NuGEN), purified using minElute Reaction Cleanup Kit (Qiagen) and quantified using a Bioanalyser 2100 with RNA 6000 Nano Kit (Agilent). cRNA was then hybridised to Human HT-12v3 expression Beadarrays (Illumina, Cambridge, United Kingdom) according to the standard protocol for NuGEN amplified samples. A new Illumina gene expression dataset was also generated from primary breast tumour core biopsies before, 10–14 days after and approximately 3 months following neoadjuvant Letrozole treatment. RNA was extracted using the miRNeasy Mini Kit with RNAse Free DNAse treatment (Qiagen). RNA was then amplified, labelled, purified, quantified and hybridised as described above for the Illumina 18 sample subset. All raw gene expression files and clinical annotation generated in this study are publicly available from the caBIG supported Edinburgh Clinical Research Facility Data Repository (https://catissuesuite.ecmc.ed.ac.uk/caarray/).

### Published MAQC and breast cancer datasets

Methods for the MAQC Illumina Human-6 Expression BeadChip (v1) and Affymetrix U133 Plus 2.0 array hybridisations are provided in the original study [18]. The NCBI GEO accession is GSE5350. Publicly available primary breast cancer datasets [26,27] were downloaded datasets from NCBI GEO and ArrayExpress. Breast cancer subtypes were assigned using three signatures from Sorlie *et al.* (2003) [5], Parker *et al.*[28] and Hu *et al*. [29] as described previously [43].

### Data processing and analysis

All data was processed using the R/Bioconductor software and packages [44], see Figure 1 for the workflow, scripts are available from the authors by request. A custom Chip Definition File (CDF) file [32] was used to map the Affymetrix data to Ensembl gene annotations and RMA implemented by the *affy* package used for normalisation. Illumina probe profiles were quantile normalised using the *lumi* package and mapped to Ensembl gene sequences using a composite list comprising mappings from reMOAT [35], BioMart and a custom BLAST sequence search of the online Ensembl gene database where there was agreement between at least two of the resources (Additional File 5). Where multiple Illumina probes represented an Ensembl gene the mean expression level was calculated. The data was then filtered using Illumina or Affymetrix probe detection P-values, removing probes that were undetected (p > 0.05 in the total minus 3 samples).

A number of batch-correction and cross-platform normalisation methods were evaluated, including mean centering [5], ComBat [21], Distance Weighted Discrimination [20] and cross-platform normalisation (XPN) [22] in order to determine the most effective method for reducing the bias imposed by the different platforms. Principal component analysis and hierarchical clustering analysis was performed using Cluster [45]. Significance analysis of Microarrays (SAM) [23] pairwise differential gene expression analysis was performed using the *siggenes* package (R/Bioconductor).

We applied a linear additive model to log-scale expression data to estimate the variances in the MAQC dataset. The variation introduced at a given level propagates additively throughout subsequent levels, allowing these variance contributions to be modelled. The total variance for a given gene was assumed to be the aggregate of individual contributions from the inter-sample, -platform, -laboratory, and -replicate variability. These contributions are assumed to be independent and randomly drawn from log-normal distributions and, as all factors meet in unique combinations a nested variance model is individually applied to each gene such that the model of the measured expression, *X*_*ijkl*_, of each probe is defined as *X*_*ijkl*_ *= μ + A*_*i*_ *+ B*_*ij*_ *+ C*_*ijk*_ *+ D*_*ijkl*_ *+* ϵ_ijkl_.(i = 1,… ,s; j = 1,…,t; k = 1,…,u; l = 1,…,v) where *μ* is the geometric-mean expression of the gene from the given sample-type, *A*_*i*_ is the effect attributed to the *i*^th^ sample, *B*_*ij*_ is the random effect of the j^th^ platform, *C*_*ijk*_ is the random effect of the k^th^ lab, *D*_*ijkl*_ is the random effect of the l^th^ replicate hybridisation, and ϵ_*ij*_ is the residual measurement error. Finally, *s* is the total number of samples, *t* is the number of platforms on which the samples were assessed, *u* is the number of labs processing the arrays, and *v* is the number of replicate samples in the corresponding platform processed in each lab. The variance of any given observation, *X*_*ijkl*_, is σ^2^_*A*_ + σ^2^_*B*_ + σ^2^_*C*_ + σ^2^_*D*_ + σ^2^; these components represent the inter-sample, inter-platform, inter-laboratory, and inter-replicate variance respectively. The estimation of σ^2^_*A*_ σ^2^_*B*_, σ^2^_*C*_ , σ^2^_*D*_, and σ^2^ is performed independently for each gene as stated in [46]. Models of this kind are formally defined in [47,48] and have previously been used to optimise gene-expression experimental design [49,50]. All variance estimates were performed using a REML procedure implemented in the *nlme* package in R [51,52].

## Competing interests

The authors declare that they have no competing interests.

## Authors’ contributions

AHS designed the study. AKT and AL extracted RNA and performed the microarray experiments. AKT, RRK, and AHS analysed the data. LR and JMD performed the biopsies, collected and documented the clinical material. AKT, RRK and AHS drafted the manuscript. All authors read and approved the final manuscript.

## Pre-publication history

The pre-publication history for this paper can be accessed here:

http://www.biomedcentral.com/1755-8794/5/35/prepub

## Supplementary Material

Additional file 1 **A) Boxplots showing the Pearson correlation coefficients within and between labs.** B) Plot showing the relationship between the false discovery rate and the number of genes identified comparing UHRR (A) with HBRR (B) using either 15 Affymetrix or 15 Illumina replicates or both together. C) Venn diagrams showing the overlaps between the 1000 most significant differentially expressed genes using the SAM method (each comparison is 15 ‘A’ samples versus 15 ‘B’ samples). Click here for file

Additional file 2 **A) Boxplots showing the range of Pearson correlation coefficients between 18 matched samples (including baseline, 14-day and 3 month from 6 patients) for different correction methods.** B) Affymetrix dataset and C) Illumina dataset boxplots showing the range of Pearson correlation coefficients between all possible sample combinations for different correction methods. D) Boxplots of standard deviation for each gene across all samples from the same subgroup (baseline and 3 months) for Affymetrix and Illumina datasets independently and when combined both before and after correction with either ComBat or XPN. E) Hierarchical clustering of samples based on Pearson correlation after either ComBat or XPN correction. Colour denotes samples from the same patient, the suffixes on patient ID’s denote as follows: ‘.1’ = Baseline, ‘.2’ = 14-day and ‘.3’ = 3 months. Click here for file

Additional file 3 Venn diagrams showing the overlaps between the 1000 most significant differentially expressed genes using A) pairwise SAM analysis and B&C) non–pairwise SAM analysis with Affymetrix (Green), Illumina (Blue) and combined (Teal). Click here for file

Additional file 4 Plots showing the relationship between false discovery rate against the number of significant differentially expressed genes identified across a range values of delta using SAM analysis in Affymetrix (Desmedt) and Illumina (Nadiri) datasets independently and when combined both before and after XPN correction to identify genes differentially expressed between the basal and luminal A (A) or luminal A and luminal B subtypes (B). Click here for file

Additional file 5 **Excel workbook with lists of the overlapping Ensembl gene identifier agreement for reMOAT, BLAST and BioMART; Lists of significant differentially expressed genes from SAM analysis; List of the 500 most variable genes from Figure**5**.** Click here for file
