# Supplementary figures and images for: Direct integration of intensity-level data from Affymetrix and Illumina microarrays improves statistical power for robust reanalysis
Source: BMC Med Genomics. 2012 Aug 21;5:35. doi: 10.1186/1755-8794-5-35 (PMC3443058; doi:10.1186/1755-8794-5-35)

**A**

## Illumina

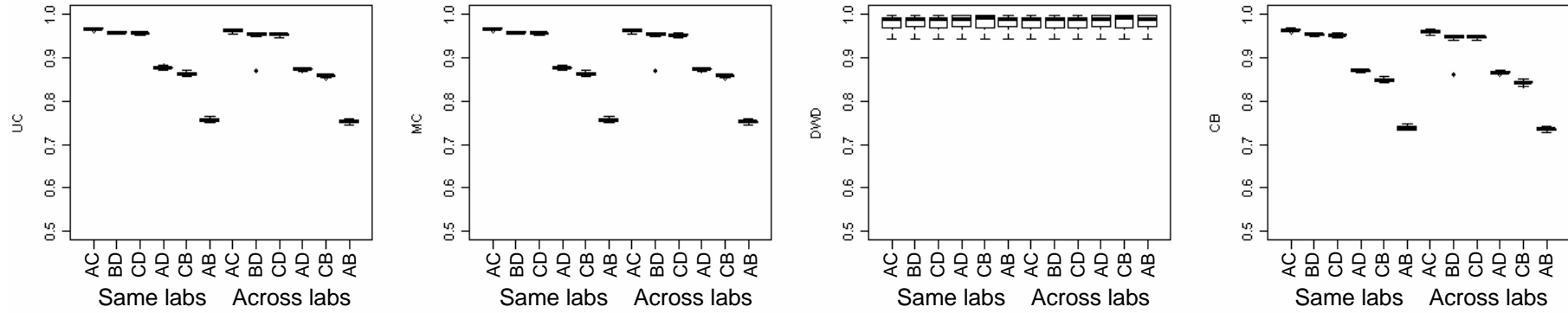

## Affymetrix

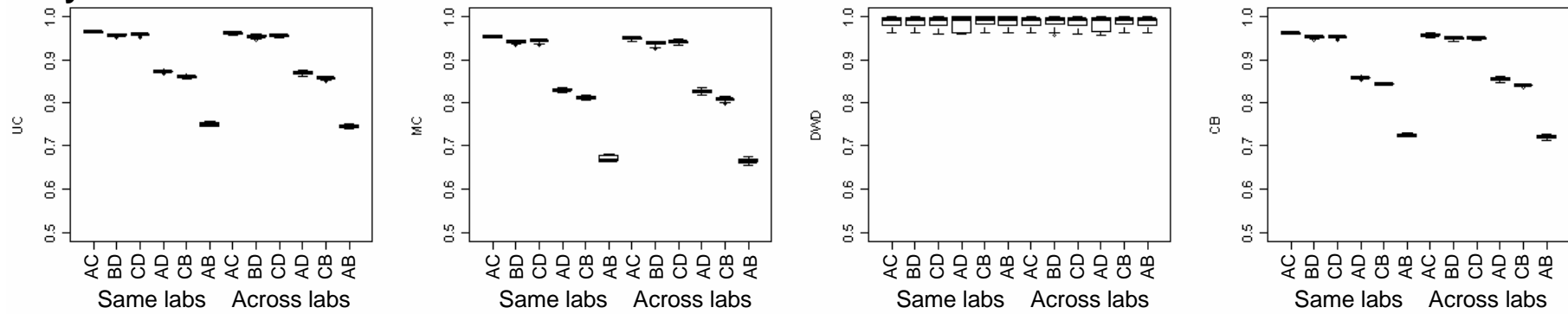

## Illumina compared to Affymetrix

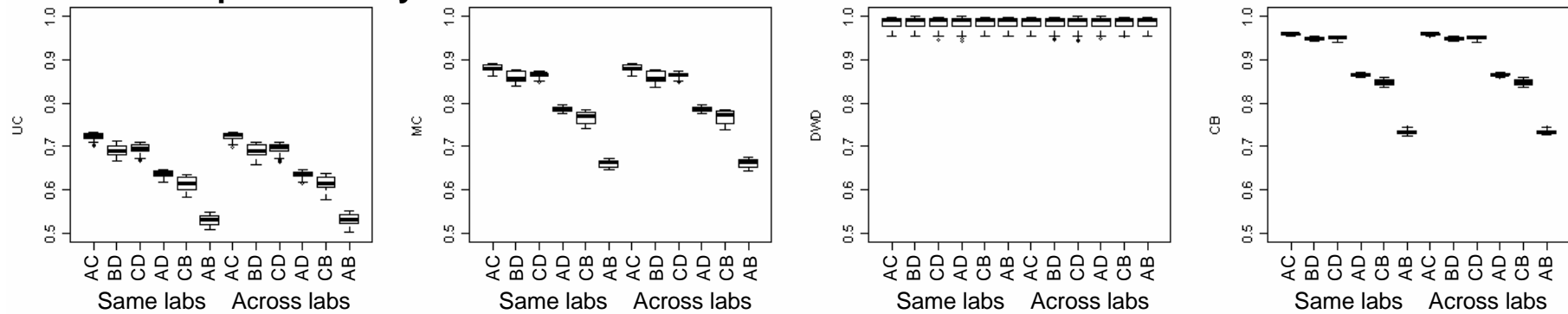

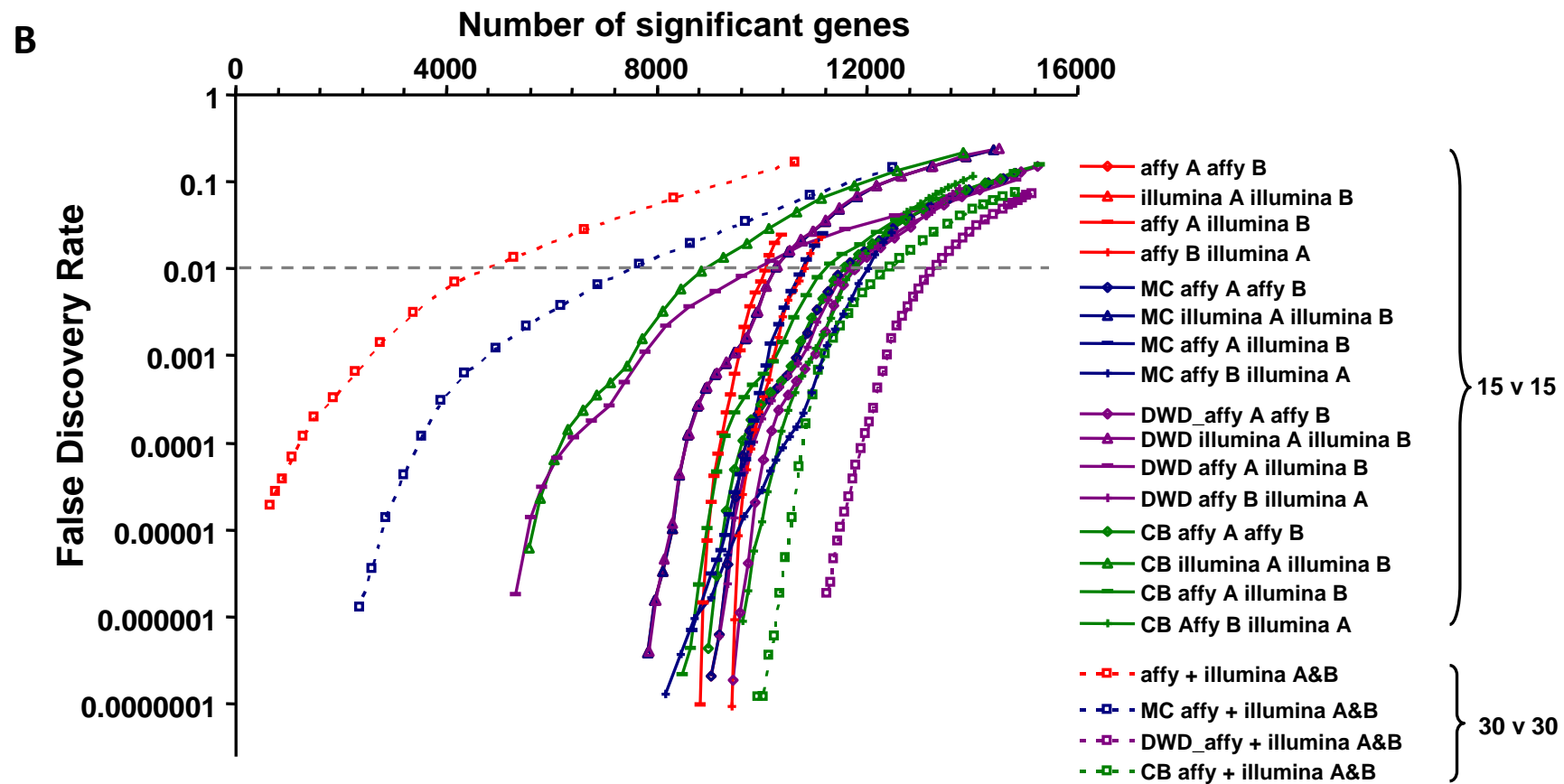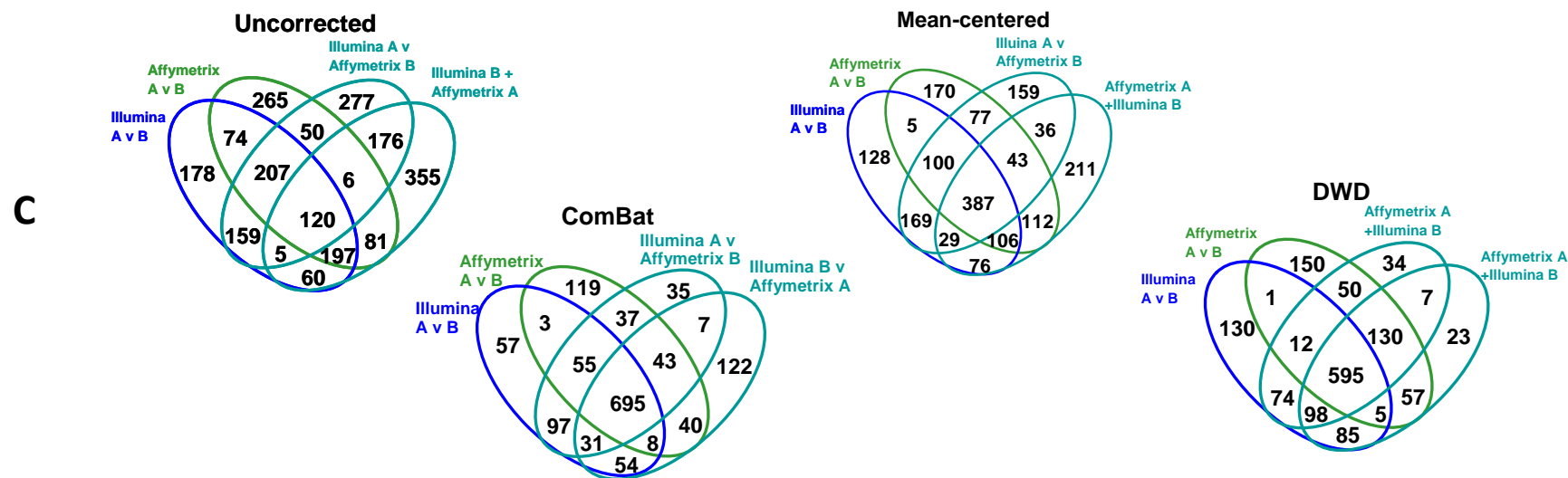

Supplement: Additional file 1 — A) Boxplots showing the Pearson correlation coefficients within and between labs. B) Plot showing the relationship between the false discovery rate and the number of genes identified comparing UHRR (A) with HBRR (B) using either 15 Affymetrix or 15 Illumina replicates or both together. C) Venn diagrams showing the overlaps between the 1000 most significant differentially expressed genes using the SAM method (each comparison is 15 ‘A’ samples versus 15 ‘B’ samples). [file 1755-8794-5-35-S1.pdf]

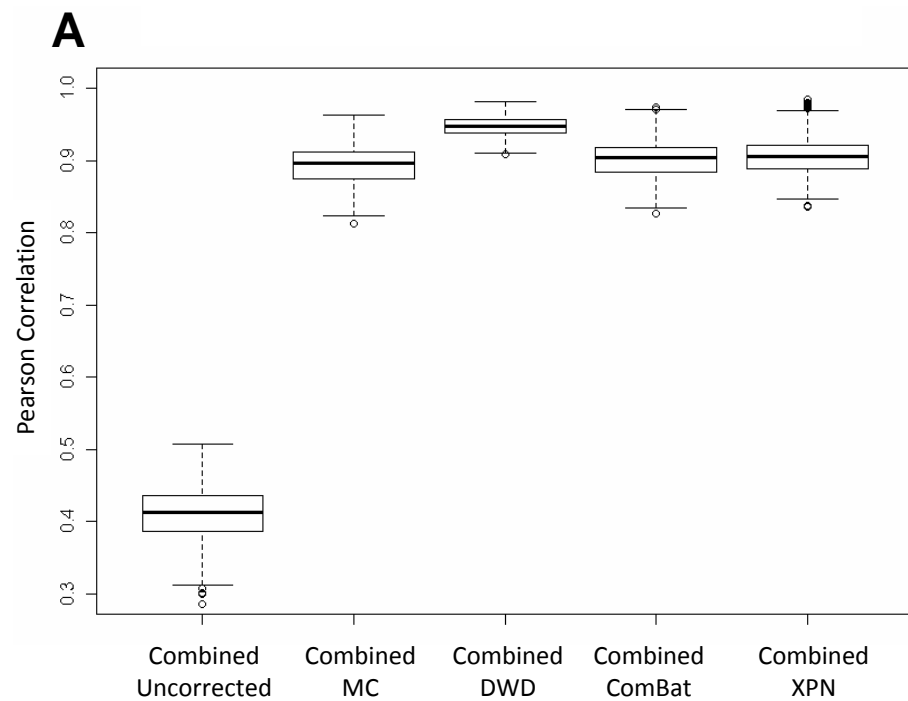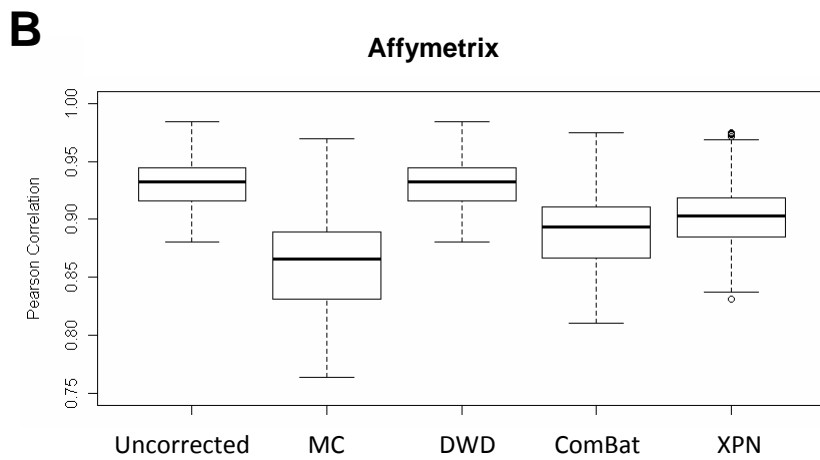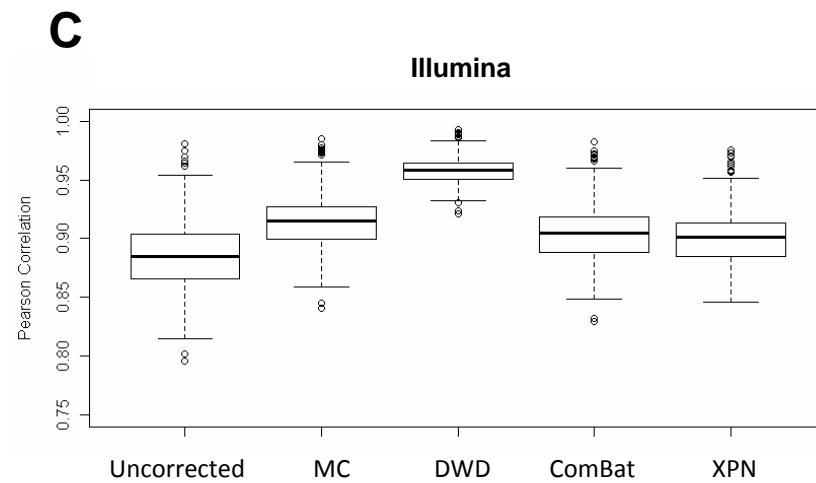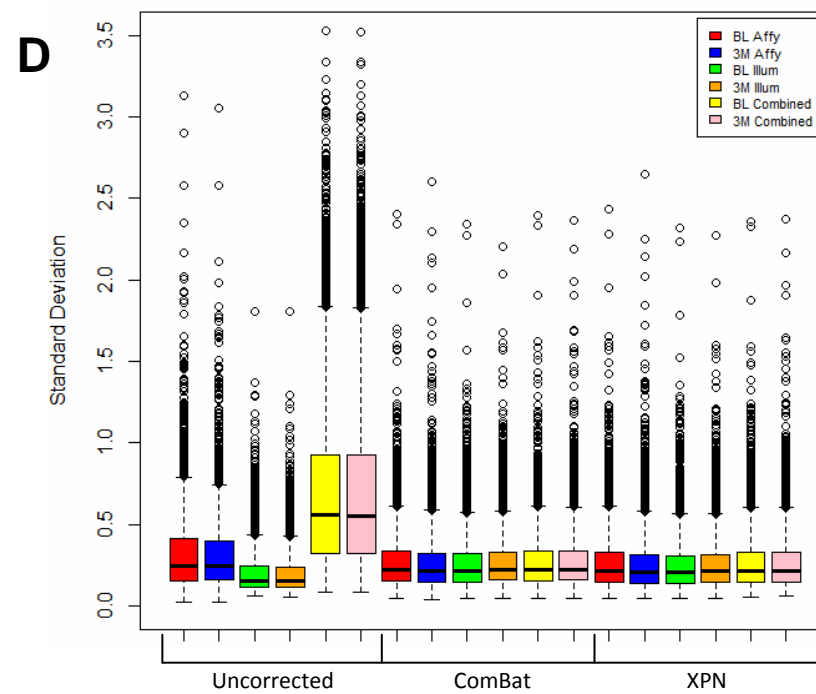

E

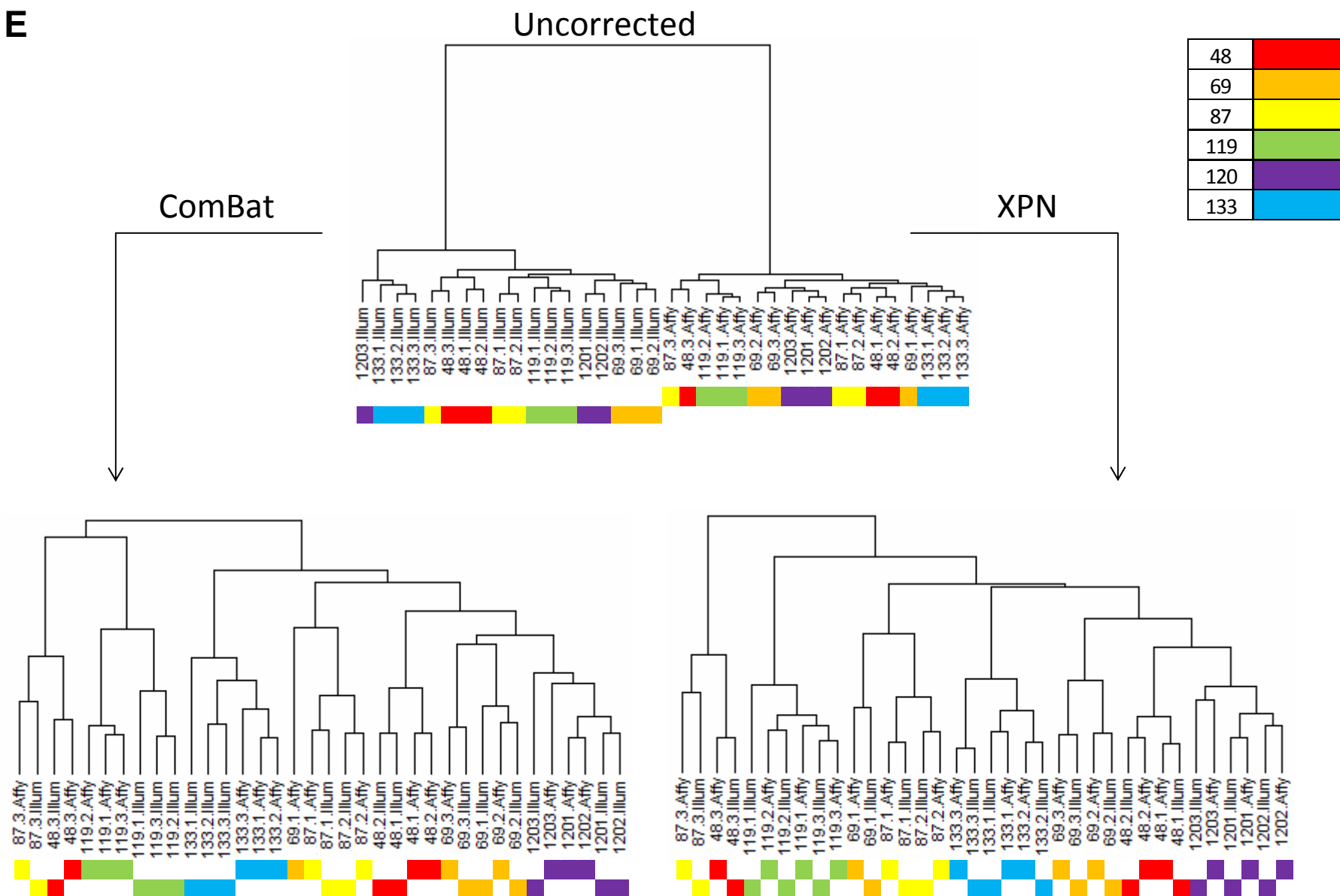

Supplement: Additional file 2 — A) Boxplots showing the range of Pearson correlation coefficients between 18 matched samples (including baseline, 14-day and 3 month from 6 patients) for different correction methods. B) Affymetrix dataset and C) Illumina dataset boxplots showing the range of Pearson correlation coefficients between all possible sample combinations for different correction methods. D) Boxplots of standard deviation for each gene across all samples from the same subgroup (baseline and 3 months) for Affymetrix and Illumina datasets independently and when combined both before and after correction with either ComBat or XPN. E) Hierarchical clustering of samples based on Pearson correlation after either ComBat or XPN correction. Colour denotes samples from the same patient, the suffixes on patient ID’s denote as follows: ‘.1’ = Baseline, ‘.2’ = 14-day and ‘.3’ = 3 months. [file 1755-8794-5-35-S2.pdf]

**A**

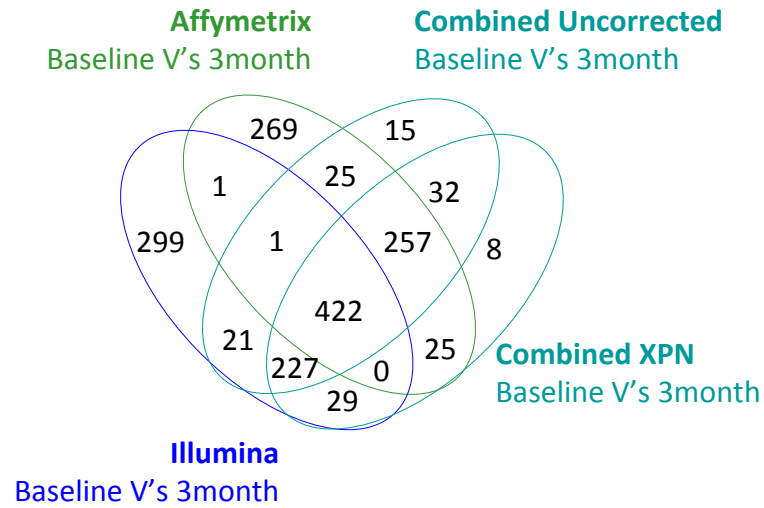

**B**

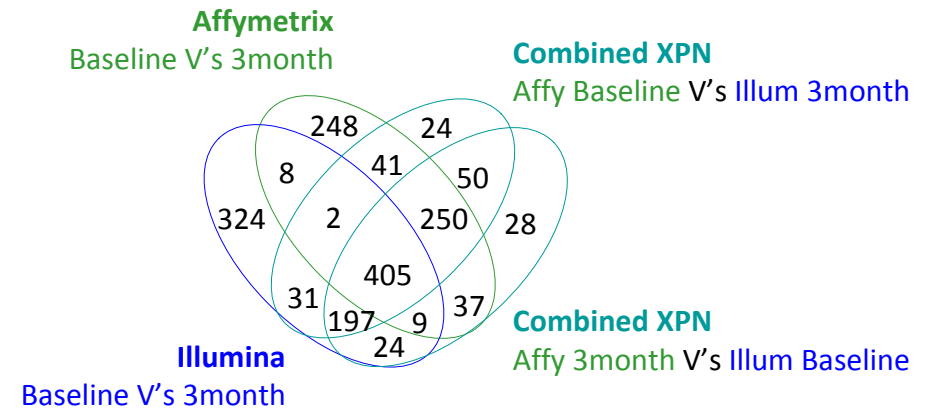

**C**

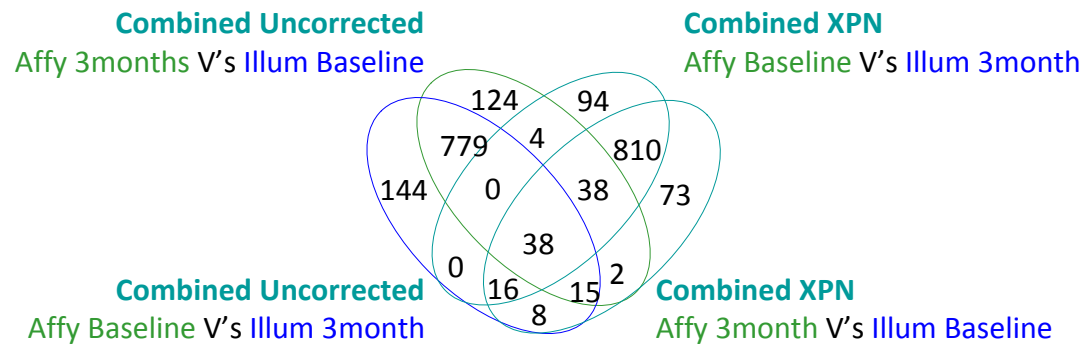

Supplement: Additional file 3 — Venn diagrams showing the overlaps between the 1000 most significant differentially expressed genes using A) pairwise SAM analysis and B&C) non–pairwise SAM analysis with Affymetrix (Green), Illumina (Blue) and combined (Teal). [file 1755-8794-5-35-S3.pdf]

**A**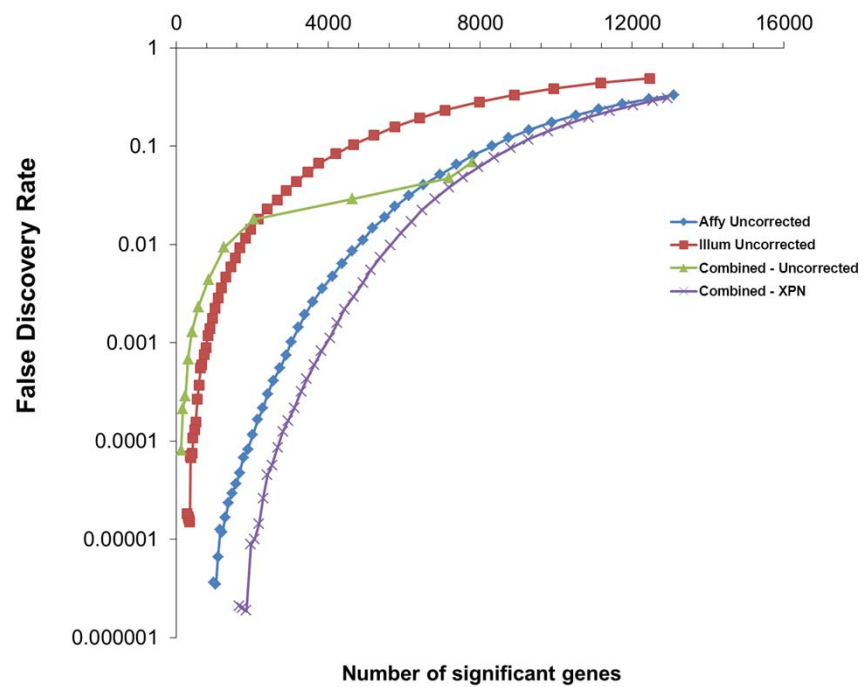**B**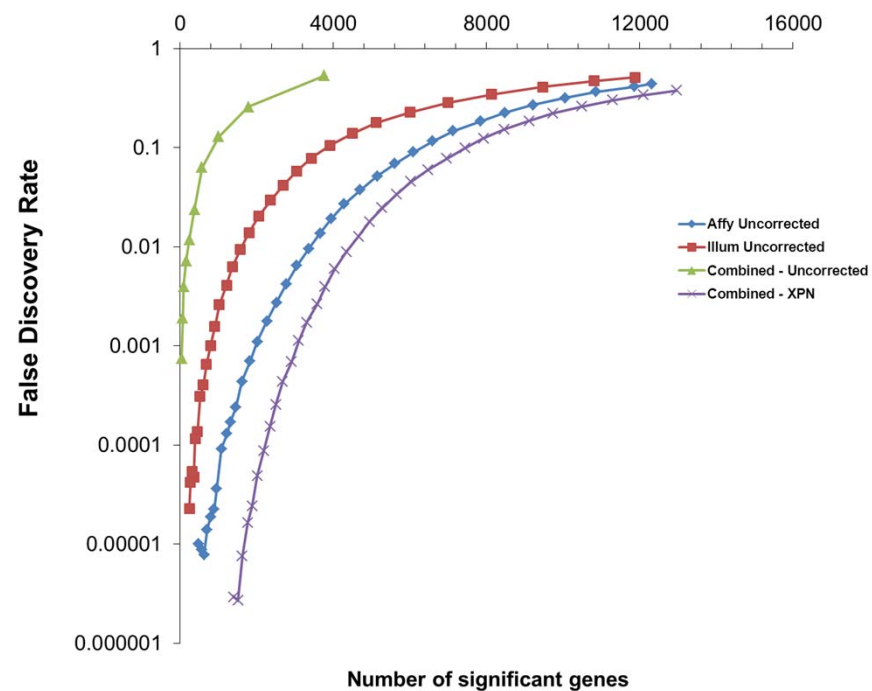

Supplement: Additional file 4 — Plots showing the relationship between false discovery rate against the number of significant differentially expressed genes identified across a range values of delta using SAM analysis in Affymetrix (Desmedt) and Illumina (Nadiri) datasets independently and when combined both before and after XPN correction to identify genes differentially expressed between the basal and luminal A (A) or luminal A and luminal B subtypes (B). [file 1755-8794-5-35-S4.pdf]
